# Supplementary material for: One-photon three-dimensional printed fused silica glass with sub-micron features
Source: Nat Commun. 2024 Mar 27;15:2689. doi: 10.1038/s41467-024-46929-x (PMC10973333; doi:10.1038/s41467-024-46929-x)
Supplement: Supplementary file 1 — Supplementary Information [file 41467_2024_46929_MOESM1_ESM.pdf]

Supplementary Information for

One-photon Three-dimensional Printed Fused Silica Glass with Sub-micron Features

Ziyong Li[1,2], Yanwen Jia[1,2,3], Ke Duan[1,2,4], Ran Xiao[1,2], Jingyu Qiao[1,2], Shuyu Liang[1,2], Shixiang Wang[5,6], Juzheng Chen[1,2], Hao Wu[1,2], Yang Lu[2,7]* and Xiewen Wen[5,6]*

[1]Department of Mechanical Engineering, City University of Hong Kong, Kowloon, Hong Kong SAR, China

[2]Nano-Manufacturing Laboratory (NML), Shenzhen Research Institute of City University of Hong Kong, Shenzhen 518057, China

[3]Department of Chemistry, Southern University of Science and Technology, Shenzhen 518055, China

[4]Department of Material Science and Engineering, College of Aerospace Science and Engineering, National University of Defense Technology, Changsha, 410073, China

[5]State Key Laboratory of Ultra-precision Machining Technology, Department of Industrial and Systems Engineering, The Hong Kong Polytechnic University, Kowloon, Hong Kong SAR, China

[6]Research Institute for Advanced Manufacturing, Department of Industrial and Systems Engineering, The Hong Kong Polytechnic University, Kowloon, Hong Kong SAR, China

[7]Department of Mechanical Engineering, The University of Hong Kong, Pokfulam Road, Hong Kong SAR, China

*Corresponding author: Xiewen Wen, e-mail: xw.wen@polyu.edu.hk; Yang Lu, e-mail: ylu1@hku.hk

This PDF file includes:

Supplementary Text

Supplementary Figures 1 to 19

Supplementary Tables 1 to 5

Supplementary References

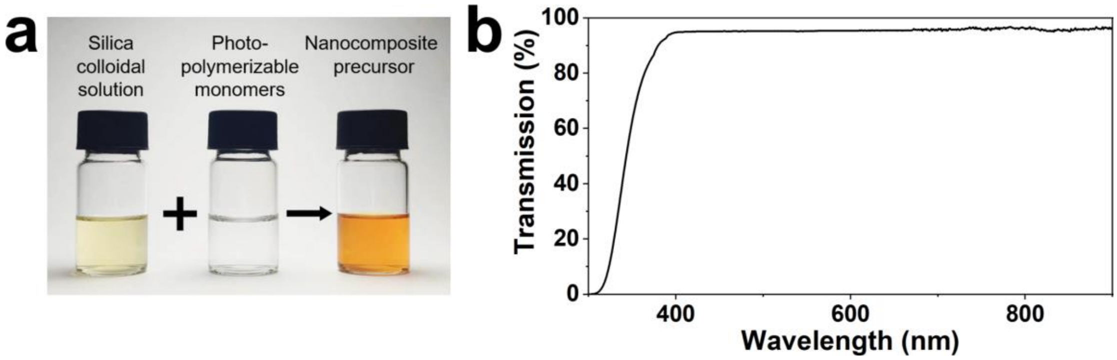

Supplementary Figure 1 | The optical transparent silica nanocomposite photo-polymerizable precursor. a Photo of the silica colloidal solution, photo-polymerizable monomers and the as-developed nanocomposite precursor. **b** UV-vis spectrum of the silica nanocomposite precursor, suggesting a transmission approximate 95% at the processing wavelength of 405 nm.

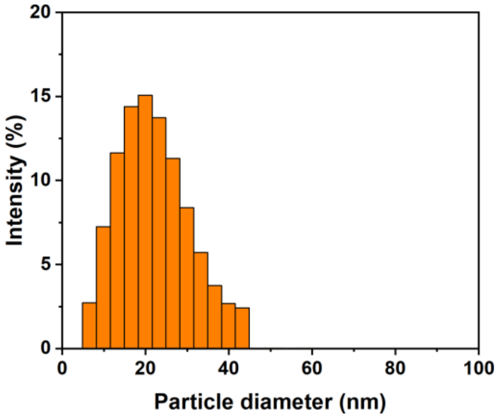

Supplementary Figure 2 | Particle diameter distribution of functionalized colloidal silica nanoparticles in polymerizable monomeric matrix.

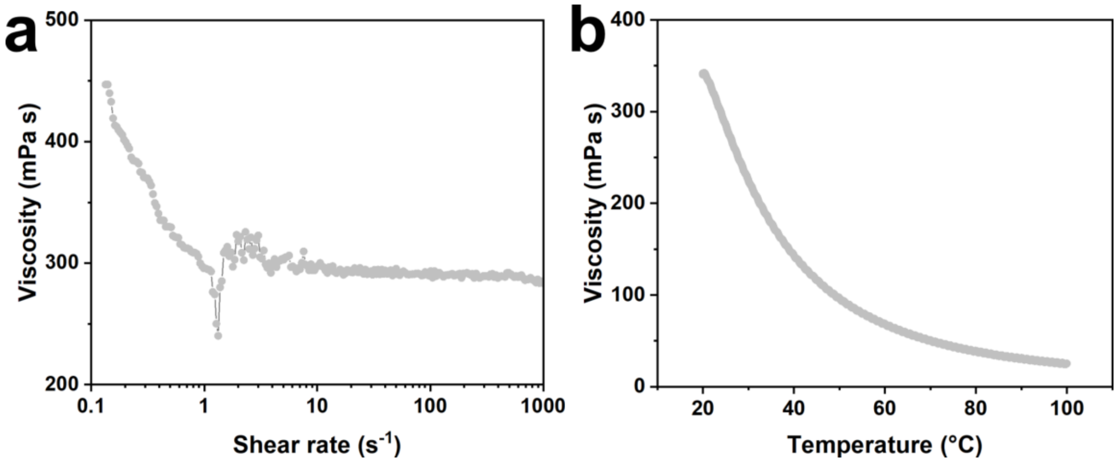

Supplementary Figure 3 | Dynamic viscosity of the silica nanocomposite photo-polymerizable precursor: viscosity versus shear rate (**a**), and viscosity versus temperature (**b**).

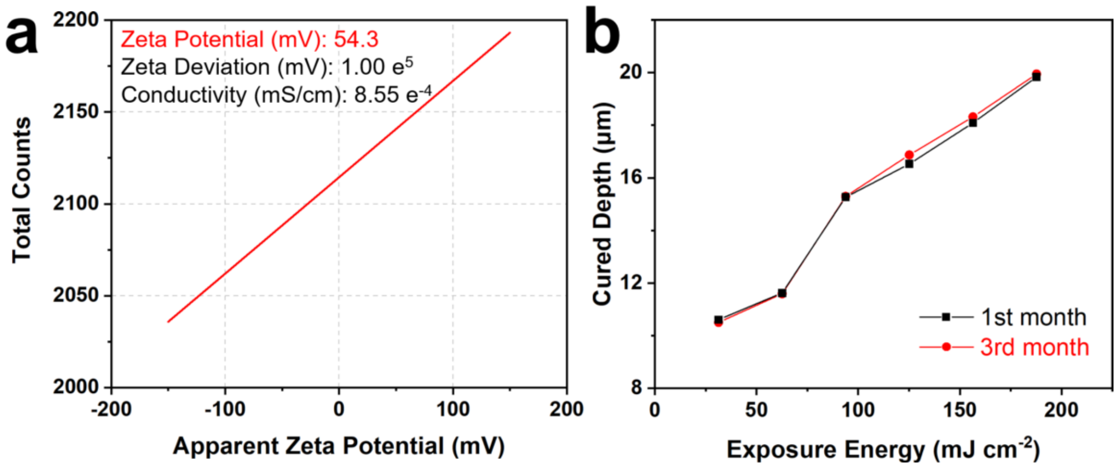

Supplementary Figure 4 | Stability characterizations of the of the silica nanocomposite photopolymerizable precursor. a Zeta potential of the silica nanocomposite photopolymerizable precursor. **b** Stereolithography cure depth (depth of a voxel upon exposure, corresponding to the penetration of the polymerization front during exposure) versus the laser power.

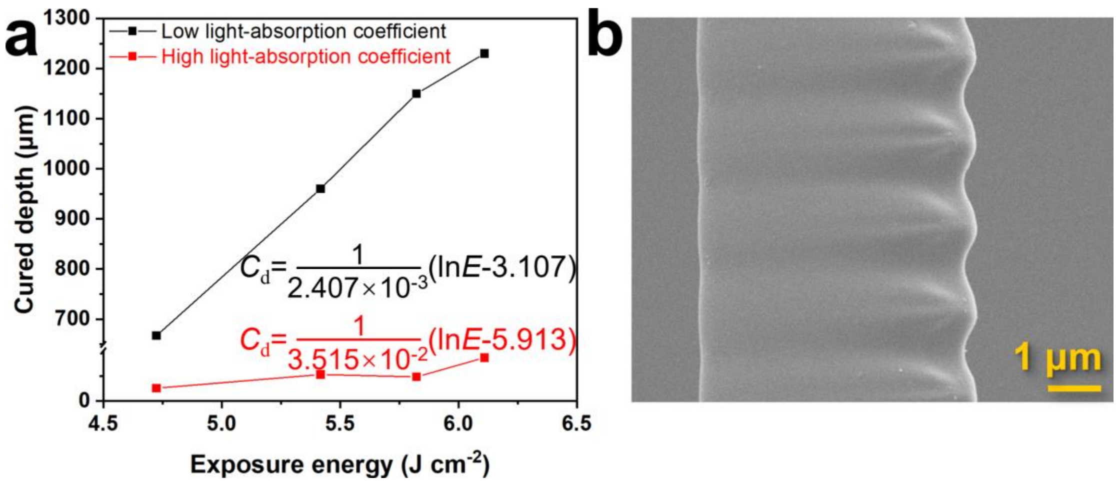

Supplementary Figure 5 | Photopolymerization properties of the silica nanocomposite photopolymerizable precursor. a The relationship between cured depth versus exposure energy of the silica nanocomposite photopolymerizable precursor with or without photo-absorber, resulting in high and low absorption coefficients, respectively. **b** Electron microscopic image of the cured thickness of the precursor with a high absorption coefficient at an exposure energy of 4.7 J cm^{-2}, and the value for the single layer is 2.78 μm.

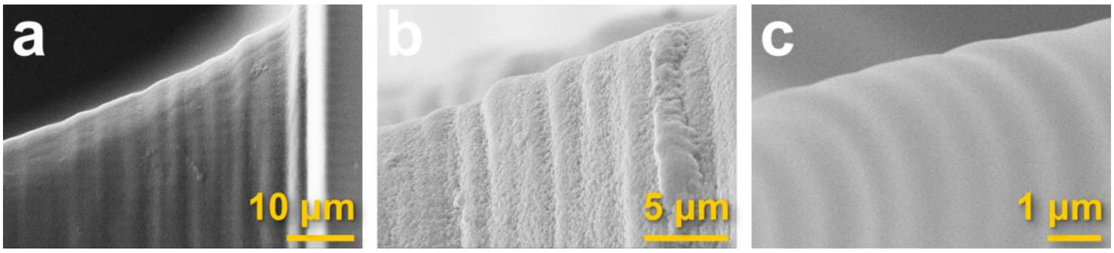

Supplementary Figure 6 | The Zoom-in SEM images of the Snake fang-inspired microneedle: as-printed (**a**), before-sintered (**b**), and after-sintered (**c**).

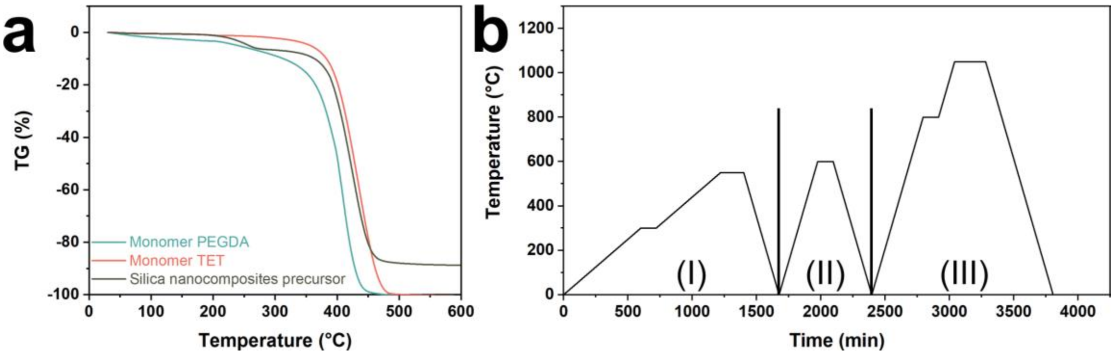

Supplementary Figure 7 | Determination of the heat treatment programme by TGA. a TGA curves for the polymerizable monomers and silica nanocomposites precursor. **b** Corresponding heating programme for: pyrolysis/debinding (I), decarbonization (II) and sintering (III) used for the composite shaped using OμSL 3D printing.

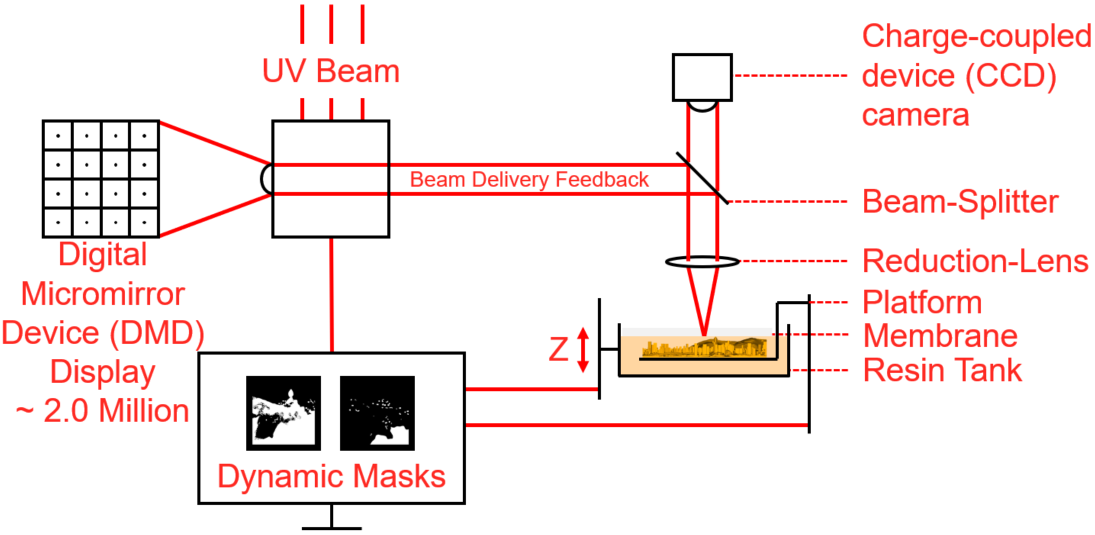

Supplementary Figure 8 | The optical setup sketch OμSL 3D printing process of miniature Hong Kong dioramas.

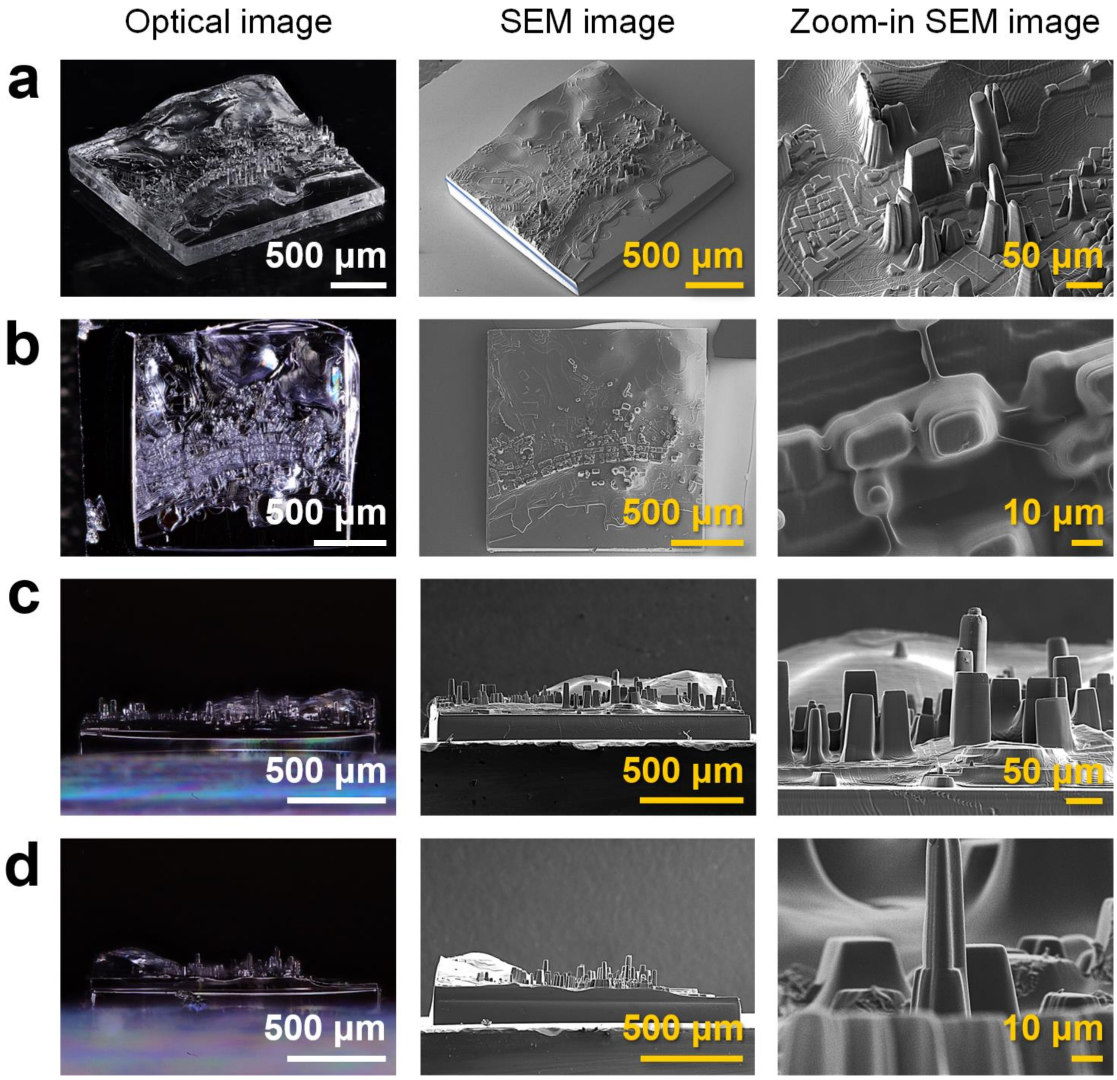

Supplementary Figure 9 | Micro-architectures of the 3D-printed transparent fused silica glass miniature Hong Kong dioramas microstructure. Optical & electron microscopic images of the perspective (**a**), vertical view (**b**), elevation view (**c**) and lateral view (**d**), respectively.

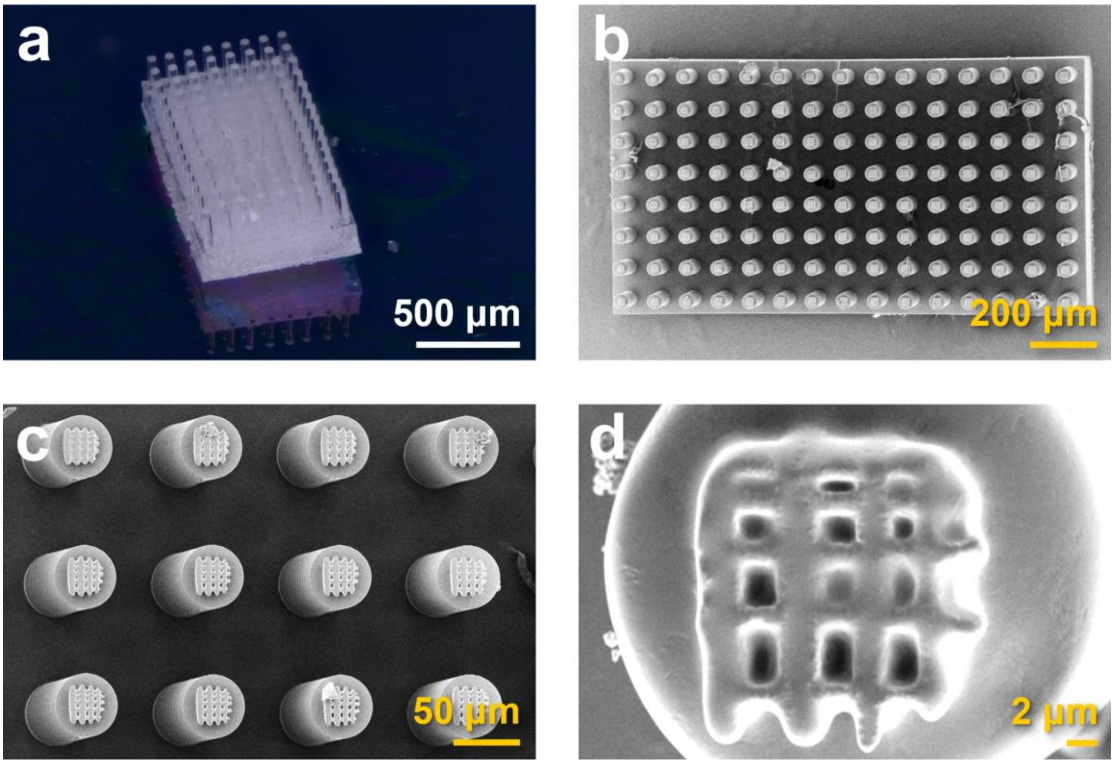

Supplementary Figure 10 | Demonstration of an OμSL 3D-printed fused silica glass structure featuring sub-micron resolution. Optical (**a**) & electron microscopic (**b**-**d**) images of a 15 × 10 column array affixed line arrays on the tops, suggesting the sub-micron features in both line width and spacing.

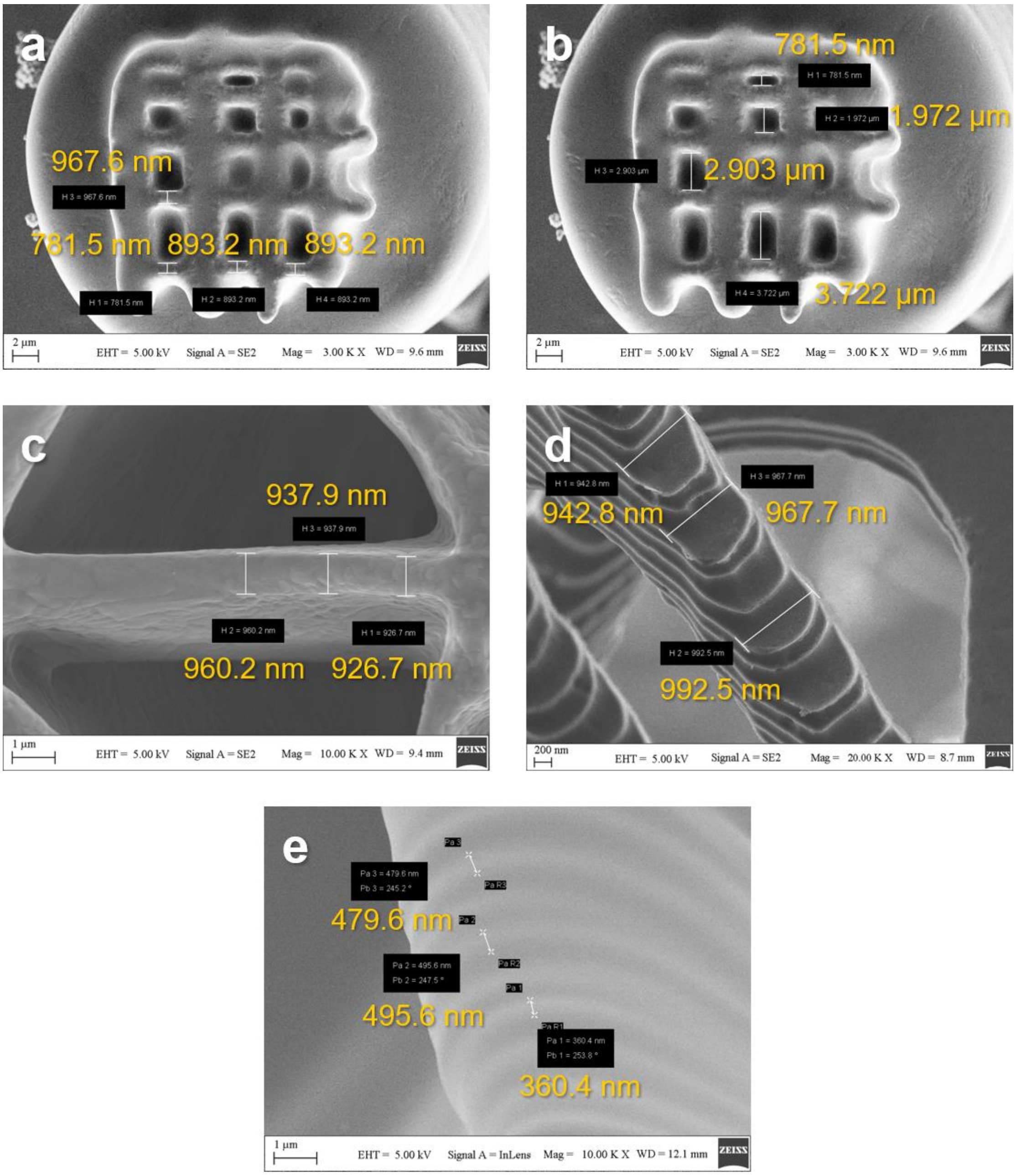

Supplementary Figure 11 | The direct measurement results obtained from SEM analysis on the OμSL 3D-printed fused silica glass sub-micron features of: line width (**a**), line spacing (**b**), thread (**c**), strut (**d**), and layer (**e**), respectively.

Supplementary Table 1 | Summary of the finest resolution against the maximum printing speed of the 3D printed transparent fused silica glass in this work and other reported technologies [1, 2, 3, 4, 5, 6, 7, 8, 9, 10, 11, 12, 13, 14].

Technology	Material	Maximum printing speed (mm^3 s^{-1})	Finest resolution (mm)
SLM[1]	Glass powders[1]	7.00E-02	5.00E-01
		2.00E-01	4.20E-01
		3.40E-01	2.80E-01
		2.10E-01	3.50E-01
		3.90E-01	7.00E-01
		2.76E-01	1.00E+00
FDM[2, 14]	Glass filaments[2]	4.60E+02	1.71E+01
		4.39E+02	2.35E+01
		6.31E+02	5.35E+01
		7.34E+02	4.61E+01
		8.03E+02	5.92E+01
	Glass filaments[14]	3.21E+00	5.45E-01
		3.13E+00	6.99E-01
		1.61E+00	6.63E-01
		2.11E+00	6.31E-01
		6.42E-01	6.44E-01
		3.19E+00	5.54E-01
		3.36E+00	6.93E-01
		4.03E+00	5.26E-01
		2.85E+00	4.03E-01

Technology	Material	Maximum printing speed (mm^3 s^{-1})	Finest resolution (mm)
SLA[3, 12, 13]	Glassomers[3]	2.84E+01	8.00E-02
		2.35E+01	2.60E-01
		4.01E+01	2.70E-01
		5.26E+01	2.00E-01
		3.48E+01	3.00E-01
	Precursor composite[12]	4.17E+00	2.60E-01
		7.98E+00	9.76E-01
		4.59E+00	7.12E-01
		5.79E+00	6.10E-01
		5.42E+00	8.77E-01
	Nano-silica slurries[13]	2.60E+00	1.08E+00
		1.97E+00	5.96E-01
		2.75E+00	2.52E-01
TPL[4, 5, 6, 7, 8, 9]	Glassomers[4]	2.97E-05	1.40E-02
		3.79E-05	1.70E-02
		5.25E-05	1.80E-02
		4.60E-05	1.72E-02
		6.91E-05	5.60E-02
		5.46E-05	6.00E-02
	Nanocomposites[5]	6.75E-08	1.20E-04
		1.95E-07	2.00E-04
		4.02E-07	1.29E-03
		1.01E-06	5.00E-04

Technology	Material	Maximum printing speed ($mm^3 s^{-1}$)	Finest resolution (mm)
TPL[4, 5, 6, 7, 8, 9]	Nanocomposites[5]	5.98E-07	6.00E-04
		2.88E-07	4.80E-04
		3.08E-07	1.00E-03
		1.89E-07	1.02E-03
		2.46E-07	2.00E-03
	Precondensed organosilicones[6]	2.37E-06	1.10E-03
		5.65E-06	5.00E-02
		2.00E-06	1.00E-01
		2.03E-06	3.70E-01
		9.11E-06	2.20E-01
		5.96E-06	1.10E-01
	POSS-organosilicones[7]	1.11E-09	1.19E-04
		1.45E-09	1.85E-04
		2.61E-09	2.25E-04
		4.79E-09	2.69E-04
		6.69E-09	3.04E-04
		8.05E-09	3.33E-04
		6.74E-09	3.66E-04
		8.27E-09	3.71E-04
		1.25E-08	4.04E-04
		1.34E-08	4.45E-04
		1.08E-08	4.72E-04
		1.17E-08	5.01E-04

Technology	Material	Maximum printing speed (mm^3 s^{-1})	Finest resolution (mm)
TPL[4, 5, 6, 7, 8, 9]	POSS-organosilicones[7]	1.47E-08	5.18E-04
		2.07E-08	5.45E-04
		2.79E-08	6.20E-04
		1.80E-08	6.54E-04
		3.26E-08	6.89E-04
		3.69E-08	7.21E-04
		3.26E-08	7.43E-04
		4.10E-08	8.86E-04
		5.03E-08	9.32E-04
		4.62E-08	9.91E-04
		1.10E-08	1.24E-04
		8.75E-09	1.46E-04
		1.66E-08	1.70E-04
		1.47E-08	1.83E-04
		2.52E-08	1.91E-04
		2.18E-08	1.96E-04
		2.53E-08	1.98E-04
		2.42E-08	2.11E-04
		1.69E-08	2.18E-04
		3.89E-08	2.25E-04
		4.91E-08	2.61E-04
		5.81E-08	2.85E-04
		3.87E-08	2.93E-04

Technology	Material	Maximum printing speed (mm^3 s^{-1})	Finest resolution (mm)
TPL[4, 5, 6, 7, 8, 9]	POSS-organosilicones[7]	5.62E-08	3.00E-04
		7.13E-08	3.12E-04
		4.66E-08	3.14E-04
		3.88E-08	3.18E-04
		6.00E-08	3.20E-04
		6.87E-08	3.22E-04
		7.00E-08	3.27E-04
		8.45E-08	3.65E-04
		5.46E-08	3.73E-04
		9.08E-08	3.84E-04
		5.88E-08	3.88E-04
		8.05E-08	4.11E-04
		6.86E-08	4.13E-04
		9.15E-08	4.11E-04
		7.77E-08	4.11E-04
		8.59E-08	4.20E-04
		8.79E-08	4.42E-04
		1.34E-07	4.64E-04
		7.52E-08	4.71E-04
		1.62E-07	4.74E-04
		9.42E-08	4.76E-04
		1.40E-07	4.90E-04
		9.47E-08	5.10E-04

Technology	Material	Maximum printing speed (mm^3 s^{-1})	Finest resolution (mm)
TPL[4, 5, 6, 7, 8, 9]	POSS-organosilicones[7]	1.06E-07	5.12E-04
		8.86E-08	5.16E-04
		1.87E-07	5.20E-04
		2.39E-07	5.80E-04
		2.24E-07	6.10E-04
		1.95E-07	6.18E-04
		1.63E-07	6.30E-04
		2.87E-07	6.37E-04
		2.24E-07	6.44E-04
		1.96E-07	6.74E-04
		2.90E-07	6.84E-04
		2.60E-07	6.91E-04
		2.26E-07	7.30E-04
		1.83E-07	7.29E-04
		3.05E-07	7.28E-04
		3.47E-07	7.77E-04
		3.75E-07	8.78E-04
		4.66E-07	9.06E-04
		4.36E-07	9.98E-04
		1.07E-07	1.17E-04
		7.59E-08	1.21E-04
		9.53E-08	1.31E-04
		1.33E-07	1.35E-04

Technology	Material	Maximum printing speed (mm^3 s^{-1})	Finest resolution (mm)
TPL[4, 5, 6, 7, 8, 9]	POSS-organosilicones[7]	6.59E-08	1.50E-04
		6.46E-08	1.48E-04
		8.74E-08	1.46E-04
		7.85E-08	1.44E-04
		6.53E-08	1.52E-04
		1.33E-07	1.60E-04
		9.44E-08	1.68E-04
		1.67E-07	1.72E-04
		8.54E-08	1.67E-04
		1.72E-07	1.71E-04
		1.63E-07	1.68E-04
		9.21E-08	1.78E-04
		2.62E-07	1.91E-04
		2.51E-07	1.99E-04
		2.83E-07	1.91E-04
		2.17E-07	2.11E-04
		2.06E-07	2.17E-04
		1.13E-07	2.13E-04
		1.07E-07	2.12E-04
		1.57E-07	2.22E-04
		1.61E-07	2.30E-04
		1.29E-07	2.25E-04
	Hydrogen silsesquioxanes[8]	1.69E-17	6.50E-05

Technology	Material	Maximum printing speed ($mm^3 s^{-1}$)	Finest resolution (mm)
TPL[4, 5, 6, 7, 8, 9]	Hydrogen silsesquioxanes[8]	1.96E-16	5.00E-04
		4.05E-16	5.40E-04
		2.50E-16	5.00E-04
		1.49E-17	6.50E-05
		1.57E-17	6.50E-05
		6.76E-17	1.30E-04
		6.60E-17	1.30E-04
		6.32E-17	1.30E-04
		1.30E-16	1.95E-04
		1.33E-16	1.95E-04
		1.12E-16	1.95E-04
	PDMS[9]	2.14E-07	8.00E-04
		3.71E-07	4.56E-03
		2.97E-07	6.08E-03
		2.29E-07	3.80E-03
CAL[10]	Glassomers[10]	3.74E+01	5.00E-01
		4.28E+00	2.00E-01
		2.26E+00	5.00E-02
		2.60E+00	7.50E-02
		3.14E+00	6.00E-02
		3.69E+00	2.50E-01
		1.44E+00	5.00E-01
		2.47E+00	1.00E-01

Technology	Material	Maximum printing speed ($mm^3 s^{-1}$)	Finest resolution (mm)
CAL[10]	Glassomers[10]	3.38E+00	8.50E-01
		3.95E+00	1.50E-01
		1.79E+00	3.50E-01
		1.06E+00	2.15E-01
DIW[11]	Silica nanoparticle gels[11]	3.72E+00	6.10E-01
		1.19E+01	5.50E-01
		9.70E+00	6.70E-01
		8.93E+00	6.90E-01
		6.57E+00	8.00E-01
		8.40E+00	1.00E+00
OμSL (this work)	Nanocomposite precursor	2.83E-02	5.59E-03
		6.63E-02	9.62E-03
		1.40E-01	3.93E-03
		3.38E-02	2.58E-03
		2.54E-02	1.15E-03
		1.12E-01	7.37E-03
		1.66E-02	8.60E-04
		2.81E-02	9.30E-04

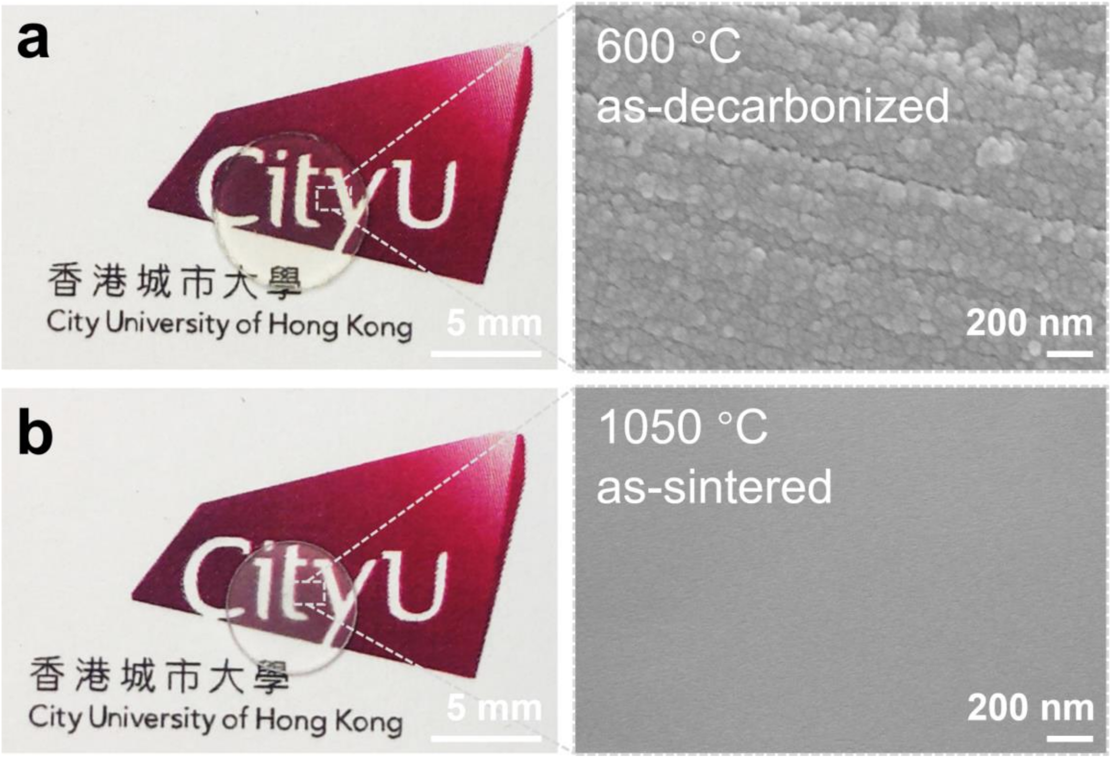

Supplementary Figure 12 | Optical photos of 3D-printed silica monoliths and corresponding SEM images of the fracture surfaces: after decarbonization at 600 °C (**a**), and after sintering at 1050 °C (**b**), respectively.

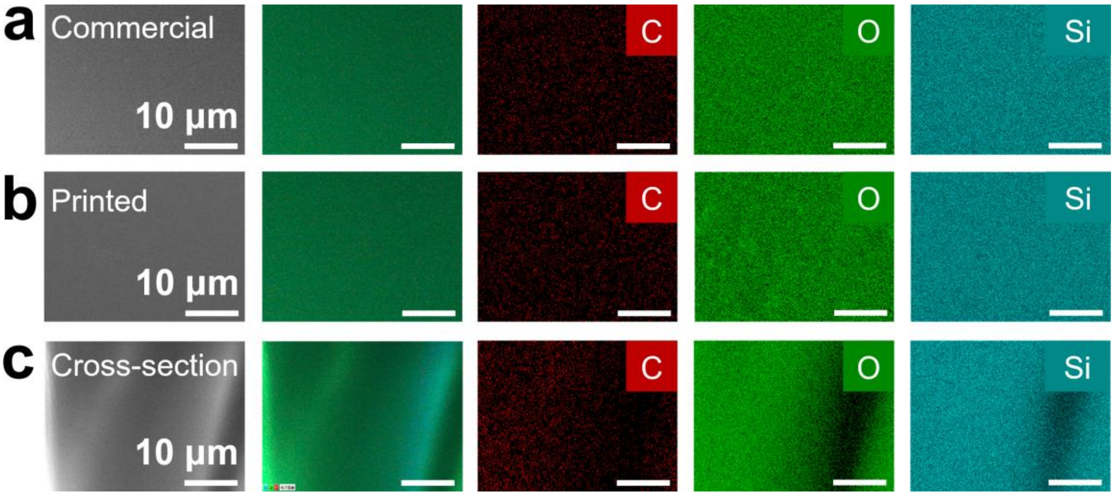

Supplementary Figure 13 | Electron microscopic and EDS mapping of: the commercial (**a**) and OμSL 3D-printed fused silica glass (surface (**b**) & cross-section (**c**)); the elements are C, O and Si, respectively.

1st (ZEISS SUPRA 55):

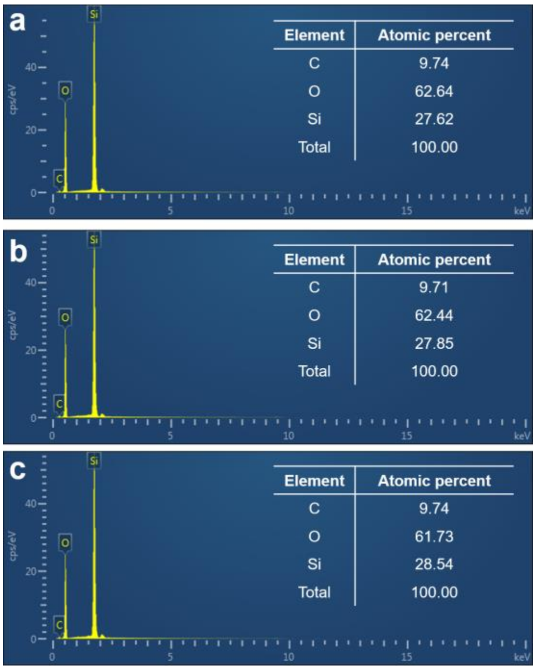

2nd (ZEISS SUPRA 55):

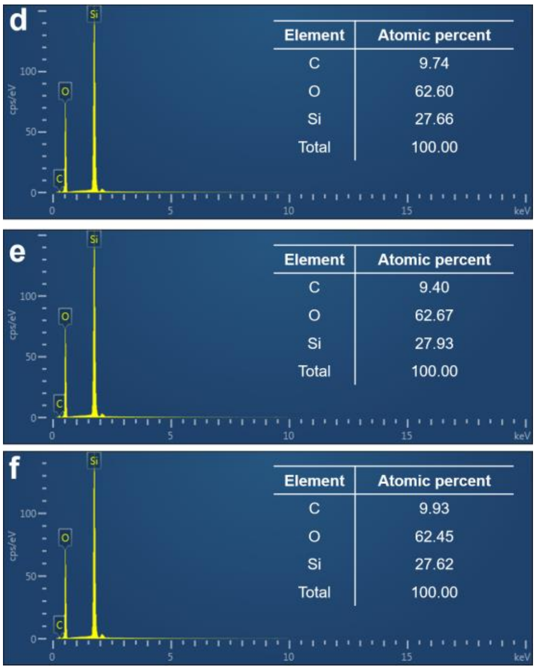

3rd (Phenom Pro):

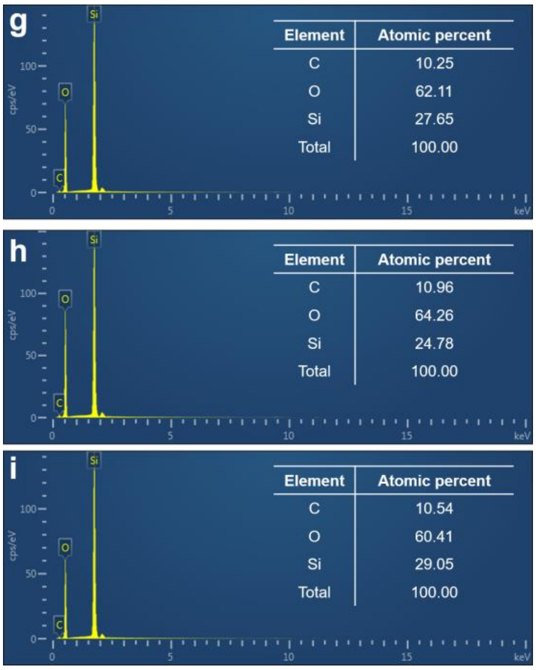

4th (FEI Quanta 450 FEG):

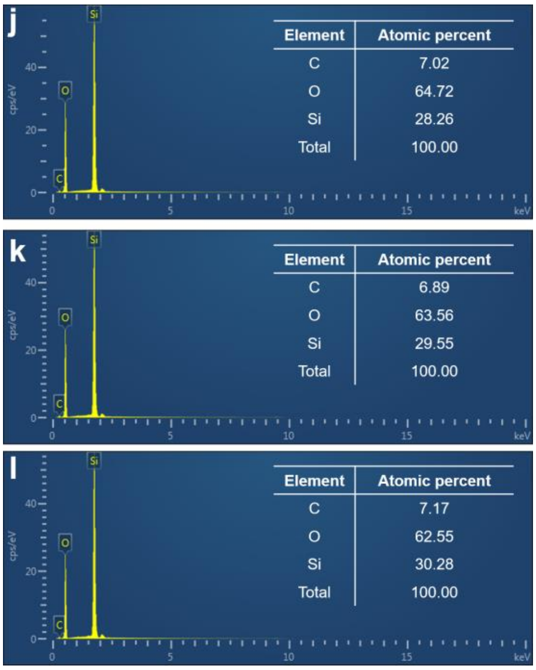

Supplementary Figure 14 | SEM-EDS spectra of: the commercial (**a**, **d**, **g** & **j**) and OμSL 3D-printed fused silica glass (surface (**b**, **e**, **h** & **k**) & cross-section (**c**, **f**, **i** & **l**)).

Supplementary Table 2 | The atomic percentages obtained from the EDS results of commercial and printed fused silica glass monoliths

Element	Atomic percent, at. %											
	Commercial				Printed				Cross-section (Printed)			
	1st	2nd	3rd	4th	1st	2nd	3rd	4th	1st	2nd	3rd	4th
C	9.74	9.74	10.25	7.02	9.71	9.40	10.25	6.89	9.74	9.93	10.54	7.17
O	62.64	62.60	62.11	64.72	62.44	62.67	62.11	63.56	61.73	62.45	60.41	62.55
Si	27.62	27.66	27.65	28.26	27.85	27.93	27.65	29.55	28.54	27.62	29.05	30.28
Total	100											

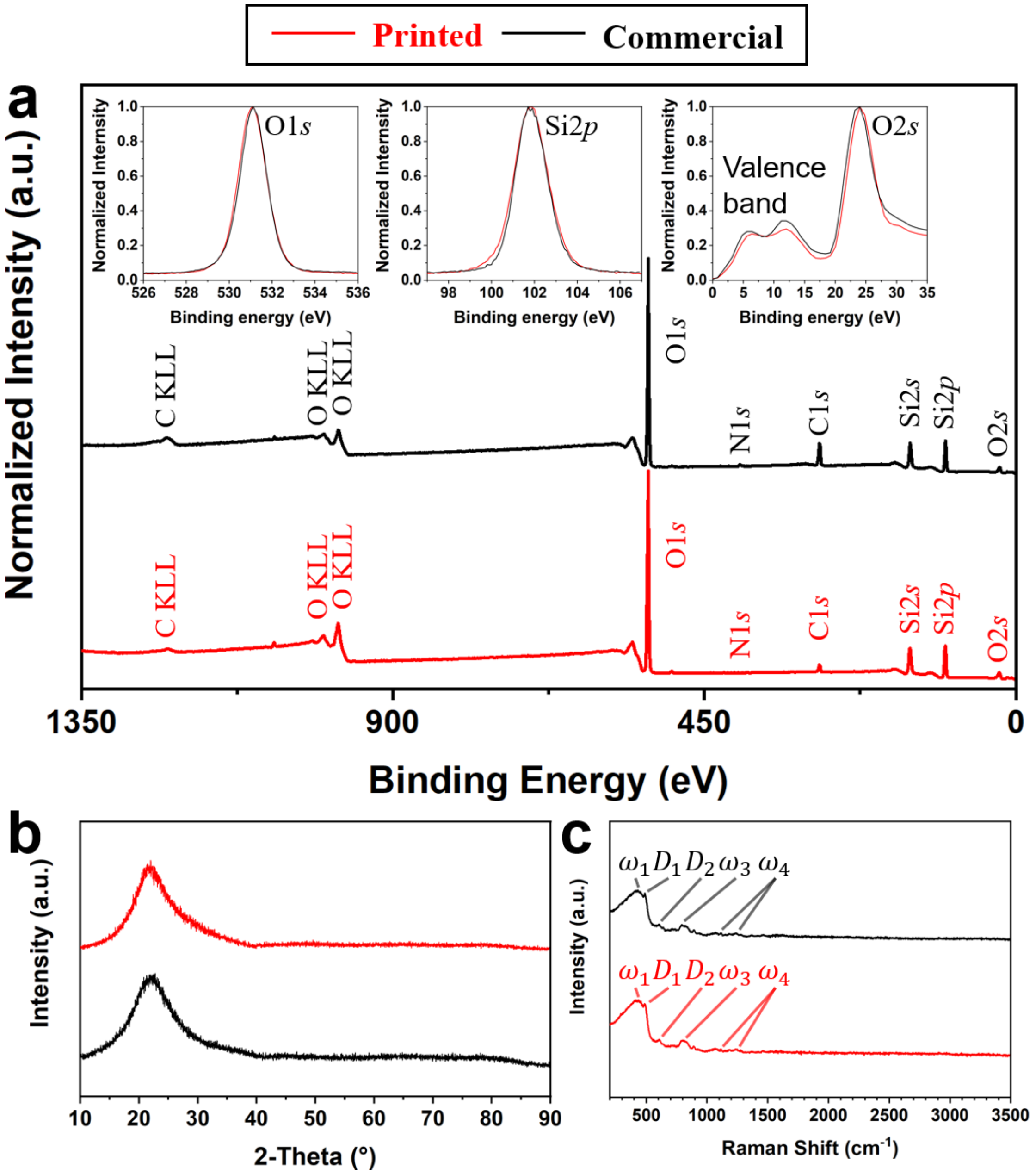

Supplementary Figure 15 | Characterization of printed-sintered fused silica glass compared with its commercial counterpart: full XPS spectrum and fine XPS spectra of each characteristic element (**a**), XRD spectrum (**b**), and Raman spectrum (**c**), respectively.

Supplementary Table 3 | The stoichiometric ratios derived from the XPS spectra of commercial and printed fused silica glass

	Atomic composition, at. %			
	O	Si	C	N
Commercial	60.35	31.24	7.30	1.11
Printed	62.86	31.15	5.10	0.89

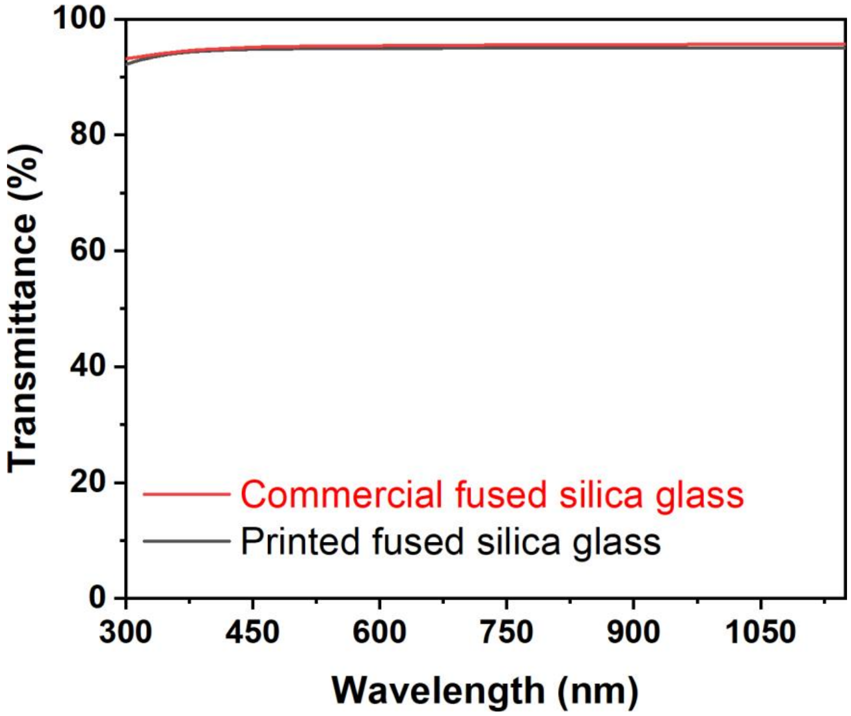

Supplementary Figure 16 | UV-Vis transmission spectra of a printed fused silica glass monolith with diameter of 5 mm and a thickness of 0.5 mm.

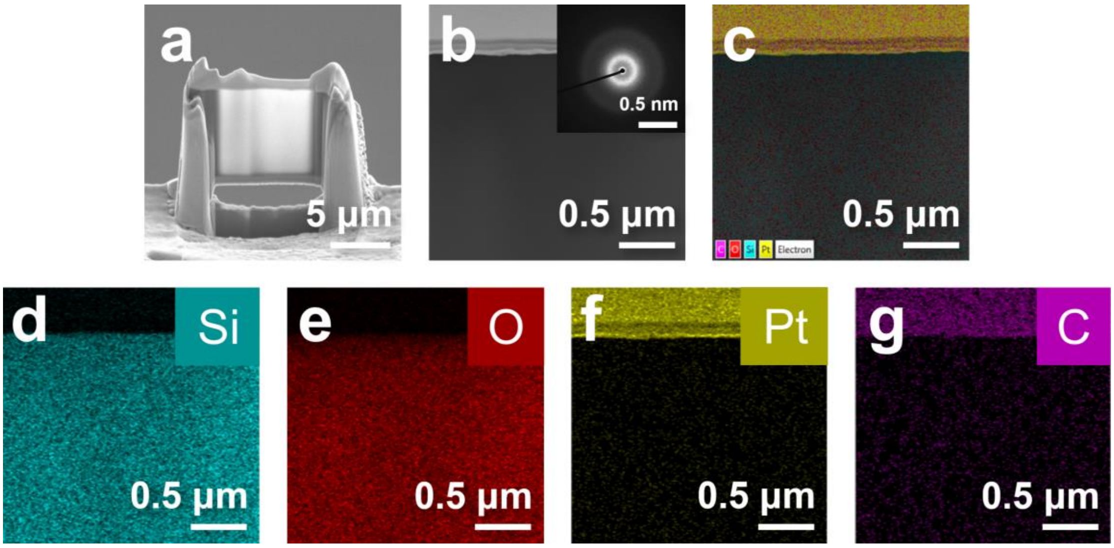

Supplementary Figure 17 | FIB-TEM characterization of the OμSL 3D-printed fused silica glass: a The printed fused silica glass after Focus Ion Beam (FIB) milling. **b** TEM image and electron diffraction pattern of the printed fused silica glass. EDS mappings of total (**c**), Si (**d**), O (**e**), Pt (**f**), and C element (**g**), respectively.

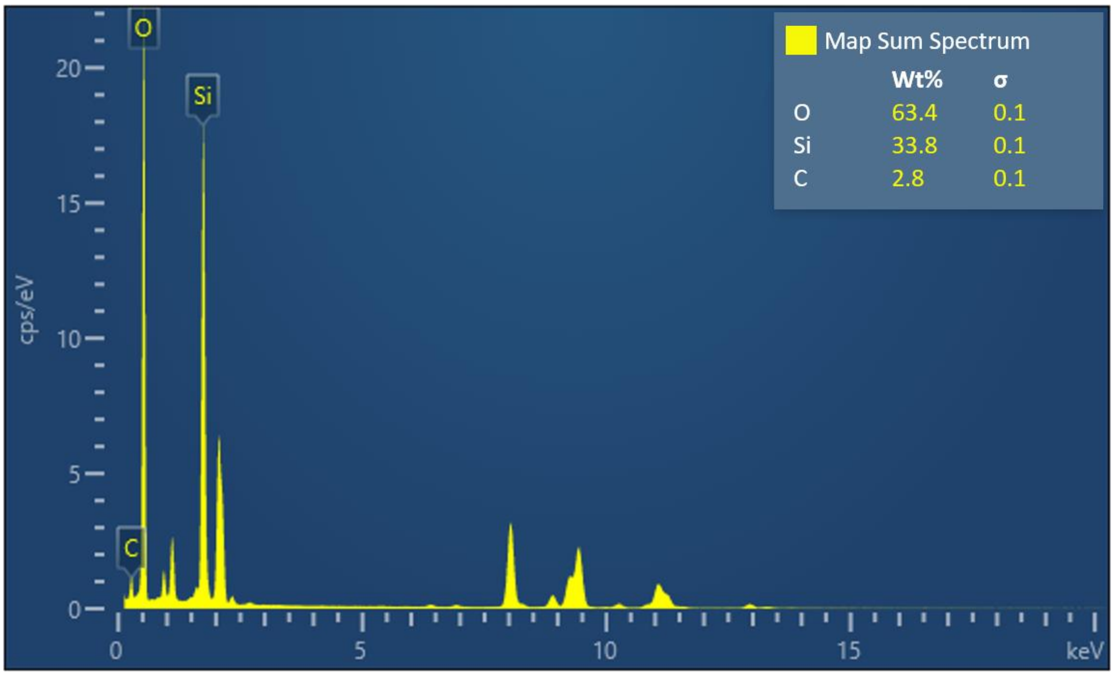

Supplementary Figure 18 | TEM-EDS spectrum of the OμSL 3D-printed fused silica glass.

Supplementary Table 4 | Parameters of OμSL 3D-printed fused silica glass microlattices

Number	Density (g cm^{-3})	Strut diameter (μm)	Maximum strength (MPa)
1	0.2774	14.61	37.33
2	0.2670	13.62	36.56
3	0.2696	13.08	40.85
4	0.3158	16.40	32.83
5	0.3156	16.39	31.05
6	0.3040	15.79	35.02

Supplementary Table 5 | Summary of the compressive strength against density of the 3D printed fused silica glass microlattice in this work and other reported high-temperature architected materials [15, 16, 17, 18, 19, 20, 21, 22, 23, 24]

Category	Density (g cm^{-2})	Strength (MPa)
SiOC microlattice[15]	3.04E-01	1.91E+01
SiC foam[16]	2.74E-01	1.31E+00
	4.00E-01	3.32E+00
	4.47E-01	3.76E+00
	4.50E-01	3.85E+00
	5.00E-01	7.30E+00
	5.26E-01	8.45E+00
	5.50E-01	8.98E+00
	5.58E-01	9.90E+00
	4.00E-01	3.19E+00
	4.50E-01	3.43E+00
	5.00E-01	4.79E+00
	5.50E-01	7.76E+00
	4.00E-01	6.01E+00
	4.50E-01	6.60E+00
	5.00E-01	9.30E+00
	5.50E-01	1.02E+01
	4.00E-01	2.98E+00
	4.50E-01	3.33E+00
	5.00E-01	5.06E+00
	5.50E-01	8.57E+00

Category	Density (g cm^{-2})	Strength (MPa)
SiC foam[16]	4.00E-01	2.33E+00
	4.50E-01	2.88E+00
	5.00E-01	4.31E+00
	5.50E-01	5.61E+00
Al-SiOC foam[17]	2.50E-01	1.38E+00
	4.00E-01	4.23E+00
	6.00E-02	9.40E+00
	2.20E-01	1.66E+01
Al_2O_3 honeycomb[18]	1.76E-01	5.90E-01
	1.61E-01	1.32E+00
	1.58E-01	1.39E+00
	1.55E-01	3.52E-01
	1.63E-01	1.00E+00
Al-Ti-SiOC microlattice[19]	1.70E+00	8.64E-02
	1.90E+00	1.08E-01
	2.10E+00	1.56E-01
	1.73E+00	6.50E-02
	1.95E+00	9.04E-02
	2.04E+00	1.24E-01
ZrO_2-SiO_2 honeycomb[20]	1.18E+00	2.06E+02
	1.14E+00	1.60E+02
	1.04E+00	1.50E+02
	1.16E+00	2.07E+02

Category	Density (g cm^{-2})	Strength (MPa)
ZrO_2-SiO_2 honeycomb[20]	1.14E+00	2.11E+02
	1.08E+00	1.74E+02
	1.19E+00	1.72E+02
	1.14E+00	1.51E+02
	1.16E+00	1.75E+02
	1.14E+00	1.69E+02
	1.18E+00	1.83E+02
	1.12E+00	1.69E+02
SiC honeycomb[21]	8.49E-01	9.30E+00
	8.97E-01	1.28E+01
	9.65E-01	9.80E+00
	8.91E-01	9.90E+00
Al_2O_3 nanolattice[22]	7.24E-03	4.55E-03
	7.86E-03	9.09E-03
	1.07E-02	1.54E-02
	1.09E-02	1.91E-02
	1.35E-02	3.71E-02
	1.77E-02	4.23E-02
	2.27E-02	6.27E-02
	1.87E-02	6.90E-02
	2.27E-02	7.63E-02
	2.33E-02	9.23E-02
	2.64E-02	1.27E-01

Category	Density (g cm^{-2})	Strength (MPa)
Al_2O_3 nanolattice[22]	2.54E-02	1.46E-01
	2.64E-02	1.78E-01
	3.21E-02	1.75E-01
	4.05E-02	2.10E-01
	4.29E-02	2.60E-01
	3.26E-02	2.35E-01
	4.46E-02	3.14E-01
	3.99E-02	3.38E-01
	5.17E-02	4.06E-01
	4.91E-02	4.52E-01
	5.41E-02	4.98E-01
	5.79E-02	5.92E-01
	6.06E-02	7.32E-01
	5.10E-02	5.92E-01
	5.41E-02	6.95E-01
	5.33E-02	8.35E-01
	7.70E-02	1.36E+00
	8.18E-02	1.62E+00
	9.64E-02	2.37E+00
	1.02E-01	2.84E+00
	1.21E-01	3.80E+00
	1.33E-01	4.50E+00
	1.52E-01	6.42E+00

Category	Density (g cm^{-2})	Strength (MPa)
Al_2O_3 nanolattice[22]	1.63E-01	7.43E+00
Al_2O_3-C nanolattice[23]	1.53E-01	2.30E+00
	1.66E-01	6.00E+00
	1.17E-01	1.90E+00
	1.27E-01	4.50E+00
	8.20E-02	4.00E-01
	1.17E-01	3.60E+00
	1.52E-01	7.60E+00
	1.74E-01	1.30E+00
	2.39E-01	1.10E+01
ZrOC microlattice[24]	1.27E-01	3.00E-01
	1.64E-01	7.57E-01
	1.97E-01	1.15E+00
	2.21E-01	2.73E+00
Fused silica glass microlattice (this work)	2.77E-01	3.73E+01
	2.67E-01	3.66E+01
	2.80E-01	4.09E+01
	3.16E-01	3.28E+01
	3.16E-01	3.11E+01
	3.04E-01	3.50E+01

Supplementary Notes

Supplementary Note 1. Demonstrations of OμSL 3D-printed fused silica glass microfluidic devices.

Fused silica glass embedded with arbitrary hollow microstructures can also be directly generated by OμSL 3D printing to a precision of tens of microns for the applications of flow cytometry [25], miniaturized chemical reactor [26] as well as high-quality optical waveguides [27]. Compared with the fused silica glass microfluidics reported by other advanced manufacturing techniques to date [25, 28, 29], OμSL printed fused silica glass microfluidics simultaneously offers high precision, straightforward process, excellent visibility, large constructive size, and freedom of sophisticate 3D designs. As demonstrations, we prepare a Y-junction microfluidic chip (Supplementary Fig. 19a) and a Tesla microfluidic valve cascade (Supplementary Fig. 19b). The Y-junction microfluidic chip consists of microchannel connected with two 50 × 50 μm^2 inlet branches at an angle of 45 ° to each other merging into a 50 × 50 μm^2 main channel. Two commercial dyes (red and blue) are firstly diluted with water then individually injected into each branch of the Y-junction, in which the testing area is pre-wetted using the deionized water. Contrary to the macro fluids determined by the inertial forces, the microfluids with low Reynolds number are dominated by viscous forces, of which laminar flow is the most obvious feature. Supplementary Fig. 19c depicts the images of the fluids sequence observed at the mixing junction after pumping into the microchannel; mixing is not observed, with a distinguish separation of red and blue fluids, suggesting the laminar flow characteristic of the fluid at microscale. The OμSL

3D-printed fused silica glass microfluidics promises to enable a wide range of applications in separation/mixture for biomedicine, synthesis/analysis in flow-through chemistry as well as Lab-on-a-chip devices in optics and photonics.

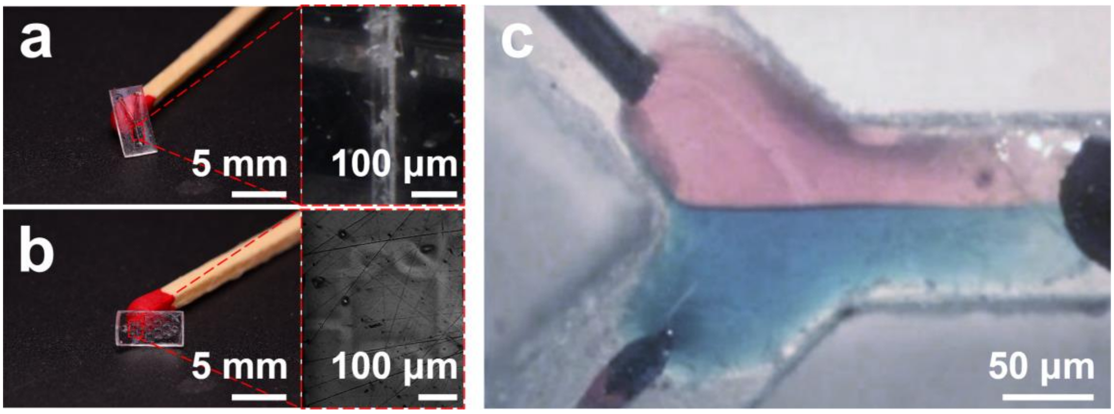

Supplementary Figure 19 | OμSL 3D-printed fused silica glass microfluidics. a A fused silica glass microfluidic chip with a Y-junction channel. The channel is of a width of 50 μm (see inset). **b** A fused silica glass microfluidic chip with a Tesla mixer cascade channel. The channel is of a width of 50 μm (see inset). **c** The flow profile in Y-junction channel, suggesting the laminar flow characteristic of the fused silica glass microfluids.

Supplementary References

1. Luo JJ, Pan H, Kinzel EC. Additive Manufacturing of Glass. *J Manuf Sci E-T Asme* **136**, 061024 (2014).

2. Klein J*, et al.* Additive Manufacturing of Optically Transparent Glass. *3d Print Addit Manuf* **2**, 92-105 (2015).

3. Kotz F*, et al.* Three-dimensional printing of transparent fused silica glass. *Nature* **544**, 337-339 (2017).

4. Kotz F*, et al.* Two-Photon Polymerization of Nanocomposites for the Fabrication of Transparent Fused Silica Glass Microstructures. *Adv Mater* **33**, 2006341 (2021).

5. Wen XW*, et al.* 3D-printed silica with nanoscale resolution. *Nat Mater* **20**, 1506-1511 (2021).

6. Hong Z, Ye P, Loy DA, Liang R. High-Precision Printing of Complex Glass Imaging Optics with Precondensed Liquid Silica Resin. *Adv Sci* **9**, e2105595 (2022).

7. Bauer J, Crook C, Baldacchini T. A sinterless, low-temperature route to 3D print nanoscale optical-grade glass. *Science* **380**, 960-966 (2023).

8. Huang PH*, et al.* Three-dimensional printing of silica glass with sub-micrometer resolution. *Nat Commun* **14**, 3305 (2023).

9. Li M*, et al.* Low-temperature 3D printing of transparent silica glass microstructures. *Sci Adv* **9**, eadi2958 (2023).

10. Toombs JT*, et al.* Volumetric additive manufacturing of silica glass with microscale computed axial lithography. *Science* **376**, 308-312 (2022).

11. Dylla-Spears R*, et al.* 3D printed gradient index glass optics. *Sci Adv* **6**, eabc7429 (2020).

12. Xu Y, Li Y, Zheng N, Zhao Q, Xie T. Transparent origami glass. *Nat Commun* **12**, 4261 (2021).

13. Cai P, Guo L, Liu LZ, Zhang QM, Li JM, Lue QT. Rapid manufacturing of silica glass parts with complex structures through stereolithography and pressureless spark plasma sintering. *Ceram Int* **48**, 55-63 (2022).

14. Zaki RM*, et al.* Direct 3D-printing of phosphate glass by fused deposition modeling. *Mater Design* **194**, 108957 (2020).

15. Li ZY*, et al.* Additive manufacturing of lightweight and high-strength polymer-derived SiOC ceramics. *Virtual Phys Prototy* **15**, 163-177 (2020).

16. Colombo P, Hellmann JR, Shelleman DL. Mechanical properties of silicon oxycarbide ceramic foams. *J Am Ceram Soc* **84**, 2245-2251 (2001).

17. Eckel ZC, Zhou CY, Martin JH, Jacobsen AJ, Carter WB, Schaedler TA. 3D PRINTING Additive manufacturing of polymer-derived ceramics. *Science* **351**, 58-62 (2016).

18. Bird RK, LaPointe TS. Evaluation of Ceramic Honeycomb Core Compression Behavior at Room Temperature. *NASA/TM* **217802**, No. L-20221 (2013).

19. Fu YL, Xu G, Chen ZW, Liu CY, Wang DM, Lao CS. Multiple metals doped polymer-derived SiOC ceramics for 3D printing. *Ceram Int* **44**, 11030-11038 (2018).

20. Liu G, Zhao Y, Wu G, Lu J. Origami and 4D printing of elastomer-derived ceramic structures. *Sci Adv* **4**, eaat0641 (2018).

21. Agrafiotis CC*, et al.* Evaluation of porous silicon carbide monolithic honeycombs as volumetric receivers/collectors of concentrated solar radiation. *Sol Energ Mat Sol C* **91**, 474-488 (2007).

22. Meza LR, Das S, Greer JR. Strong, lightweight, and recoverable three-dimensional ceramic nanolattices. *Science* **345**, 1322-1326 (2014).

23. Bauer J, Hengsbach S, Tesari I, Schwaiger R, Kraft O. High-strength cellular ceramic composites with 3D microarchitecture. *P Natl Acad Sci USA* **111**, 2453-2458 (2014).

24. Fu YL, Chen ZW, Xu G, Wei Y, Lao CS. Preparation and stereolithography 3D printing of ultralight and ultrastrong ZrOC porous ceramics. *J Alloy Compd* **789**, 867-873 (2019).

25. Kotz F, *et al.* Fabrication of arbitrary three-dimensional suspended hollow microstructures in transparent fused silica glass. *Nat Commun* **10**, 1439 (2019).

26. Elvira KS, Solvas XCI, Wootton RCR, deMello AJ. The past, present and potential for microfluidic reactor technology in chemical synthesis. *Nat Chem* **5**, 905-915 (2013).

27. Okamoto K. Progress and technical challenge for planar waveguide devices: silica and silicon waveguides. *Laser Photonics Rev* **6**, 14-23 (2012).

28. Fischer J, Mueller JB, Kaschke J, Wolf TJA, Unterreiner AN, Wegener M. Three-dimensional multi-photon direct laser writing with variable repetition rate. *Opt Express* **21**, 26244-26260 (2013).

29. Shepherd RF, *et al.* Stop-Flow Lithography of Colloidal, Glass, and Silicon Microcomponents. *Adv Mater* **20**, 4734-4739 (2008).
