# Supplementary material for: One-photon three-dimensional printed fused silica glass with sub-micron features
Source: Nat Commun. 2024 Mar 27;15:2689. doi: 10.1038/s41467-024-46929-x (PMC10973333; doi:10.1038/s41467-024-46929-x)
Supplement: Supplementary file 3 — Description of Additional Supplementary Files [file 41467_2024_46929_MOESM3_ESM.pdf]

**Description of Additional Supplementary Files**

**File Name: Supplementary Movie 1**
**Description:** The in-situ compression behavior of the OμSL 3D-printed fused silica glass microlattice. The mechanical metamaterial underwent a sequential process of linear elastic deformation, localized failure of unit struts, and final failure along its shear band.

**File Name: Supplementary Movie 2**
**Description:** The non-loss droplet transportation experiment on the OμSL 3D-printed fused silica glass superhydrophobic micro-surface. A 3 μL deionized water droplet adhering to the top surface was successfully transported without loss to the bottom surface.

**File Name: Supplementary Movie 3**
**Description:** The droplets coalescence experiment on the OμSL 3D-printed fused silica glass superhydrophobic micro-surfaces. The two 2 μL droplets of deionized water adhered to the textured surfaces, successively contacted, co-dissolved, and coalesced at the bottom without incurring any loss.

**File Name: Supplementary Movie 4**
**Description:** The utilization of OμSL 3D-printed fused silica glass superhydrophobic micro-surfaces as the droplet micro-reactor for aggressive chemicals. A 2 μL droplet of 0.5 mol L-1 CuSO4 was pre-adhered to the micro-surface followed by depositing another 0.5 mol L-1 NaOH droplet. The droplet maintained its spherical shape and remained perched on the microstructure throughout the whole reaction.
